# A pilot study on the evaluation of postural strategies in young and elderly subjects using a tridimensional electromagnetic system

**DOI:** 10.5935/1808-8694.20130038

**Published:** 2015-11-02

**Authors:** José Ailton Oliveira Carneiro, Taiza Elaine Grespan Santos-Pontelli, José Fernando Colafêmina, Antonio Adilton Oliveira Carneiro, Eduardo Ferriolli

**Affiliations:** a PhD. Professor at the Physical Education Program - State University of Southeastern Bahia - UESB; b PhD - Department of Neurosciences and Behavior, Medical School of Ribeirão Preto-FMRP/USP (Post-doctoral student); c PhD - Department of Ophthalmology, Otorhinolaryngology, and Head and Neck Surgery, Medical School of Ribeirão Preto/FMRP-USP (Professor); d PhD - Department of Physics and Mathematics, School of Philosophy, Sciences, and Literature of Ribeirão Preto/FFCLRP-USP (Professor); e PhD (Professor - Department of General Practice, Medical School of Ribeirão Preto - FMRP/USP). Medical School of Ribeirão Preto/FMRP-USP

**Keywords:** aged, postural balance, sensory deprivation, young adult

## Abstract

**Abstract:**

One resorts to various postural strategies while attempting to maintain balance.

**Objective:**

To assess the postural strategies adopted by young and elderly subjects in varying sensory conditions by using a system of tridimensional electromagnetic sensors positioned on the projection of the first thoracic vertebra and on the sacral region. Postural oscillation values for young and elderly subjects were also reported.

**Method:**

This observational cross-sectional study enrolled 25 young and 16 elderly individuals. A Polhemus™ device equipped with two sensors was used to assess postural oscillation parameters (maximum displacement, mean velocity, and trajectory). Data acquisition was carried out with subjects standing while undergoing a 90-second test in four sensory conditions: eyes opened, eyes closed, on a stable surface, and on an unstable surface.

**Results:**

Sensors 1 and 2 presented significant cross-correlations in all sensory conditions for both groups (r > 0.99; *p* < 0.001). No statistically significant differences were seen when the cross-correlations for both groups were compared.

**Conclusion:**

This study presented an important tool to analyze postural oscillation and assess the postural strategies of young and elderly subjects in different sensory conditions. Young and elderly individuals presented strong correlations between sensors (ankle strategy), but no statistically significant differences were seen between groups.

## INTRODUCTION

The postural control system must be robust enough to regulate balance under unstable conditions and versatile enough to allow a rapid initiation of movement. When standing still, the human being is not immobile, but oscillates. These oscillations with linear or angular movements of the body are neuromuscular responses used for the maintenance of postural balance^1^, ^2^. When instabilities occur, the nervous system must generate, with both anticipation and immediacy, coordinated responses to maintain postural balance^3^. The integrity of the Central Nervous System (CNS) is necessary for the recognition of the positions and movements of the head in relation to the body and the environment. Also, in order to maintain an adequate postural balance, the CNS depends on the afferent information of the vestibular, visual, proprioceptive and interoceptive systems, which promote the interaction of body with space^2^, ^4^, ^5^.

In order to maintain balance, some neuromuscular responses or postural strategies are commonly used by adults and two models have been proposed from the study of the biomechanical properties of static posture. The first model is known as “inverted pendulum”, where the oscillations of head and hip are concordant, as in the “ankle strategy”, where this is the oscillating articulation^6^, ^7^. The second, more flexible and characterized by discordant oscillations of head and hip, is called “double inverted pendulum” or “hip strategy”. A third strategy from the study of the biomechanical properties of dynamic posture, including the analysis of axial synergy and anticipatory postural adjustments has been proposed and is known as “step strategy”^8^, ^9^. When an external disturbance occurs, it is followed by the postural strategies described above (ankle or hip strategies) or by the dynamic step strategy^6^, ^7^.

One of the difficulties for researchers and therapists who work with postural balance is the scarcity of instruments that quantify postural oscillation more precisely. The most frequently measured variable for the evaluation of postural control is the Center of Pressure (COP). The COP is the application point of the resultant of vertical forces over the support surface, studied by the use of force platforms^1^. Other methods for posturographic analysis described in the literature include baropodometry using electronic baropodometer systems^10^ and multisegmental posturography using electromagnetic sensors^11^.

Multisegmental posturography detects and registers small body oscillations, thus allowing a direct investigation of the movement kinematics of postural control, since it may provide the analysis of various body segments according to the number of sensors. The most frequently analysed structures are ankle, hip, trunk and head^11^, ^12^, ^13^, ^14^.

The Polhemus™ electromagnetic sensor system is a portable instrument that permits assessment in a diversity of environments and is more accessible than the force platform, thus potentially representing a very important tool in this area of knowledge. Although this system has been previously employed for the analisys of body oscillations and postural strategies^11^, the sensors in that previous study were positioned on the head and lumbar region, which have high mobility. These positions of the sensors do not allow a precise study of postural strategies because the head can move independently of the trunk, and both head and lumbar region can move without hip motion.

Therefore, the objective of the present study was to evaluate the postural strategies of young and elderly subjects using the tridimensional electromagnetic system with two sensors, positioned on the projection of the first thoracic vertebra and on the sacral region. Furthermore, we reported values for postural oscillation in healthy young and elderly subjects evaluated with this equipment under different sensory conditions.

## METHOD

### Subjects

This was across-sectional study including 25 healthy young volunteers (15 women and 10 men) and 16 healthy elderly women with a mean age of 25.8 ± 4.2 and 68.3 ± 2.7 years, mean body mass of 63.9 ± 13.1 and 59.1 ± 7.1 kg mean height of 1.68 ± 0.1 and 1.58 ± 0.05 m and mean BMI of 22.6 ± 3.3 and 23.4 ± 1.6 kg.m^2^, respectively. The volunteers were interviewed for the identification of diseases. Exclusion criteria were: presence of vestibular, neurological, osteomuscular, cardiovascular, and psychiatric diseases and the presence of visual impairments without the use of corrective lenses. All volunteers received detailed information about the research and signed an informed consent form prior to participation. The study was approved by the local Human Research Ethics Committee (protocol number 244/2008).

### Instrumentation

The Polhemus™ Patriot brand electromagnetic transducer system with two sensors was employed to determine the position and spatial orientation (postural oscillation) of the segment on which it was fixed, i.e., trunk and sacral region, in three dimensions, through the registration of the relative position between the receptor and the transmitter sensors. This system, consisting of three perpendicular coils (22.9 mm × 28.3 mm × 15.2 mm) connected to an amplifier, is based on both emission and detection of magnetic fields, with a precision of 2 mm (absolute) and approximately 0.1 mm (relative), and good accuracy. The system has a normal sensitivity within a range of 1 m (adjustable to 3 m) between the receptor and the transmitter sensors. Six spatial parameters (x, y, z coordinates and Euler anglesθ, ϕ,ρ), from each sensor were acquired and transferred to a notebook in real time through a USB interface and a LabView 8.0 environment with an specially designed software. The acquisition sampling frequency was 100 Hz.

Data processing was done by scanning in parallel, allowing the visualization of the profile of voluntary oscillation in real time through the graphic presentation of the three independent coordinates x, y, z of the record. These coordinates represent the movements in the anteroposterior, mediolateral and craniocaudal axis.

The surfaces employed for assessment were a wood platform measuring 1 × 50 × 50 cm (stable surface) and a 30 kg/m^3^ foam platform measuring 5 × 50 × 50 cm (unstable surface)^15^.

### Procedure

**Figure 1:**
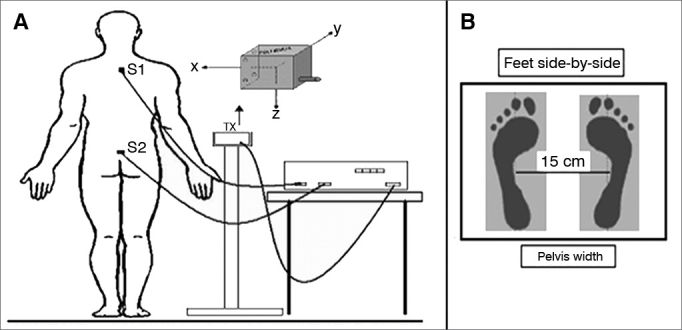
A Location of the electromagnetic sensors. Tx: Transmitter and representation of the three planes; S1 and S2: 1^st^ thoracic vertebrae and sacral region; B: Surface with position of the feet.

During evaluation, the volunteers remained standing in the orthostatic position on the wood platform (stable surface) and later on the foam platform (unstable surface). The foam platform reduces the quality and/or quantity of somatosensory information at the ankle and increases the instability of the subjects. The magnetic sensors were placed on the skin, fixed with a bandage over the spinous process of the first thoracic vertebra (S1) and over the sacral region (S2). The magnetic transmitter coil was placed on a stable support, approximately 40 cm away from the volunteer's body at intermediate sensor height (Figure 1).

Before the beginning of data acquisition, the volunteers were asked to stand with arms along the side of their body and with their feet side-by-side and parallel at the pelvis width. The researcher did not observe the presence of ante or retroversion movement of the volunteers' hips during any of the tests.

Data acquisition was performed for one trial of 90 seconds in each of the following four conditions: eyes open on a stable surface (EOSS), eyes open on an unstable surface (EOUS), eyes closed on a stable surface (ECSS), and eyes closed on an unstable surface (ECUS). During the open eye tests, the volunteers were asked to look at a wall-mounted object placed at a distance of 1 m, at eye height. This set of tests is also known as the modified Clinical Test of Sensory Interaction on Balance (mCTSIB)^2^.

### Data analysis

The parameters of postural oscillation analyzed in the present study were: maximum displacement, trajectory and mean speed. The maximum anteroposterior (AP) displacement was defined as the highest amplitude of movement at the anteroposterior axis (AP), with the maximum mediolateral displacement being the highest movement at the mediolateral axis (ML). The trajectory (total displacement) was defined as the path followed by the body during data acquisition in the AP and ML axis. The mean speed was defined as the ratio between total displacement and time.

To determine the postural strategies, whether sensor 1 and sensor 2 were in agreement, normalized cross-correlations with zero lag were calculated^16^ from the Mat-Lab program. The result obtained lies between -1 (signals identical but opposed in phase) and +1 (signals strongly identical). Furthermore, the more the correlation is close to zero, the more the signals are different. The agreement of the sensors reflects no hip motion and, therefore, ankle strategy. On the other hand, the absence of agreement of the sensors reflects hip motion and, therefore, hip strategy.

For the statistical analysis with correction of the data according to the height of each volunteer, the following calculation was performed: variable/height. The Shapiro-Wilk test was used to test whether the variables had normal distribution. Initially, descriptive statistics were used to analyze the physical characteristics of the studied population. In order to compare the sensory conditions in the same group, ANOVA and the post hoc Tukey test were applied. The *Student t* test was used to compare the differences in postural oscillation between groups. The Pearson correlation test was used to analyze correlations between variables with and without adjustment for height of the volunteers. The level of significance was two-tailed and set at α< 0.05. The data were analyzed using the SPSS software version 16.0, and version 6.0 was used for graph drawing, statistical package origin (Mi-crocal Origin®, 6.0, USA) was used.

## RESULTS

Young and elderly volunteers were studied. There was not significant difference in maximum displacement, mean speed or trajectory between young women and men. The variables with the correction for height are not presented because they showed a strong correlation (r ≥ 0.95; *p* < 0.001) with the variables without correction.

Table 1 shows the parameters of oscillation of young and elderly subjects in the open eye (OE) and closed eye (CE) conditions and on stable (SS) and unstable surfaces (US), and the results of statistical analysis of the comparisons between sensory conditions and between groups.

**Table 1:** Parameters of postural oscillation of sensors 1 and 2 of young and elderly subjects. Data are reported as means and standard deviations.

		Group	EOSS	ECSS	EOUS	ECUS
Maximum AP displacement (cm)	S1	Young	2.37 ± 0.97	2.78 ± 1.23	3.51 ± 1.47^c^	5.14 ± 2.13^b^,^d^
		Elderly	2.26 ± 1.23	2.62 ± 1.8	4.45 ± 1.90^c^	4.77 ± 2.26^d^
	S2	Young	2.53 ± 0.95	2.65 ± 0.86	3.48 ± 1.66^c^	4.40 ± 1.49^b^,^d^
		Elderly	2.59 ± 0.1	2.84 ± 1.27	4.24 ± 1.59^c^	4.88 ± 1.90^d^
Maximum ML displacement (cm)	S1	Young	1.65 ± 1.14	1.33 ± 0.58	2.28 ± 0.7^c^	3.01 ± 1.12^b^,^d^
		Elderly	1.27 ± 0.75	1.33 ± 1.11	2.71 ± 1.27^c^	3.09 ± 1.61^d^
	S2	Young	1.29 ± 0.64	1.30 ± 0.69	2.25 ± 0.94^c^	2.50 ± 0.82^b^,^d^
		Elderly	1.34 ± 0.64	1.42 ± 1.08	2.45 ± 0.96^c^	3.05 ± 1.34^d^
AP trajectory (cm)	S1	Young	76.6 ± 14.37	86.93 ± 16.09	90.45 ± 25.69	110.95 ± 27.78^b^,^d^
		Elderly	79.51 ± 14.88	86.33 ± 21.36	110.96 ± 23.53*	115.50 ± 23.62
	S2	Young	74.76 ± 17.10	77.44 ± 19.36	88.25 ± 26.23	106.10 ± 30.23^b^,^d^
		Elderly	86.05 ± 18.55	98.56 ± 31.65*	106.96 ± 23.53*	113.46 ± 23.20
ML trajectory (cm)	S1	Young	41.39 ± 15	44.7 ± 11.30	54.62 ± 11.85^c^	64.85 ± 11.45^b^,^d^
		Elderly	43.76 ± 14.44	44.44 ± 17.84	63.37 ± 23.39^c^	66.73 ± 25.31^d^
	S2	Young	42.15 ± 11.48	41.50 ± 12.72	50.43 ± 13.61	60.58 ± 15.48^b^,^d^
		Elderly	48.63 ± 16.68	51.56 ± 14.74*	63.24 ± 14.71*	66.53 ± 22.39
Total trajectory (cm)	S1	Young	118.46 ± 19	128.42 ± 21.16	142.67 ± 32.9^c^	168.68 ± 34.37^b^,^d^
		Elderly	122 ± 17.88	128.29 ± 21.64	166.24 ± 30.10^c^	173.45 ± 32.29^d^
	S2	Young	116.38 ± 23.3	120.17 ± 29.01	137.17 ± 34.3^c^	162.43 ± 40.65^b^,^d^
		Elderly	133 ± 17.29*	148.64 ± 32.76*	163.03 ± 22.67^c^,*	174.19 ± 30.79^d^
Mean AP speed (cm/s)	S1	Young	0.85 ± 0.15	0.97 ± 0.17	1.00 ± 0.28	1.23 ± 0.31^b^,^d^
		Elderly	0.88 ± 0.17	0.96 ± 0.24	1.24 ± 0.25^c^,*	1.29 ± 0.26^d^
	S2	Young	0.83 ± 0.19	0.86 ± 0.21	0.98 ± 0.29	1.18 ± 0.33^b^,^d^
		Elderly	0.96 ± 0.21	1.10 ± 0.35*	1.17 ± 0.24*	1.26 ± 0.26
Mean ML speed (cm/s)	S1	Young	0.48 ± 0.12	0.5 ± 0.12	0.60 ± 0.13^c^	0.72 ± 0.12^b^,^d^
		Elderly	0.48 ± 0.16	0.49 ± 0.2	0.71 ± 0.26^c^	0.74 ± 0.28^d^
	S2	Young	0.47 ± 0.12	0.46 ± 0.14	0.56 ± 0.15	0.67 ± 0.17^b^,^d^
		Elderly	0.54 ± 0.19	0.57 ± 0.16*	0.71 ± 0.16*	0.74 ± 0.25
Total mean speed (cm/s)	S1	Young	1.31 ± 0.21	1.43 ± 0.23	1.58 ± 0.36^c^	1.87 ± 0.38^b^,^d^
		Elderly	1.36 ± 0.20	1.43 ± 0.24	1.86 ± 0.34^c^,*	1.93 ± 0.36^d^
	S2	Young	1.29 ± 0.26	1.34 ± 0.32	1.52 ± 0.38^c^	1.81 ± 0.45^b^,^d^
		Elderly	1.48 ± 0.19*	1.66 ± 0.37*	1.80 ± 0.25^c^,*	1.94 ± 0.34^d^

AP: Anteroposterior; ML: Mediolateral; S1 and S2: Sensors 1 and 2; EOSS: Eyes open, stable surface; ECSS: Eyes closed, stable surface; EOUS: Eyes open, unstable surface; ECUS: Eyes closed, unstable surface

Signifcant difference (*p* < 0.05) between the conditions EOSS vs. ECSS infuence of vision

^b^ Between the conditions EOUS vs. ECUS infuence of vision

^c^ Between the conditions EOSS vs. EOUS infuence of surface

^d^ Between the conditions ECSS vs. ECUS infuence of surface.

^*^ Signifcant difference (*p* < 0.05) between young and elderly subjects.

In intergroup analysis, differences were found in the total mean speed and trajectory of S2 in the EOSS condition, in the AP, ML and total mean speed and trajectory of S2 in the ECSS condition, in the AP and total mean speed and trajectory of S1 and S2 and in the ML mean speed and trajectory of S2 in the EOUS condition (*p* < 0.05).

Table 2 shows the agreement of the S1 and S2 body segments, presenting high cross-correlations in all sensory conditions for both groups, with r > 0.99; *p* < 0.001. No differences in cross-correlation results were observed between groups.

**Table 2:** Cross-correlations of S1 and S2 in the different sensory conditions and comparison of the cross-correlation between groups (Mann-Whitney U test).

	Young Mean ± SD	Elderly Mean ± SD	*p*-value
EOSS	0.99 ± 0.01	0.99 ± 0.06	0.34
ECSS	0.99 ± 0.01	0.99 ± 0.06	0.83
EOUS	0.99 ± 0.04	0.99 ± 0.01	0.81
ECUS	0.99 ± 0.01	0.99 ± 0.01	0.61

**Figure 2:**
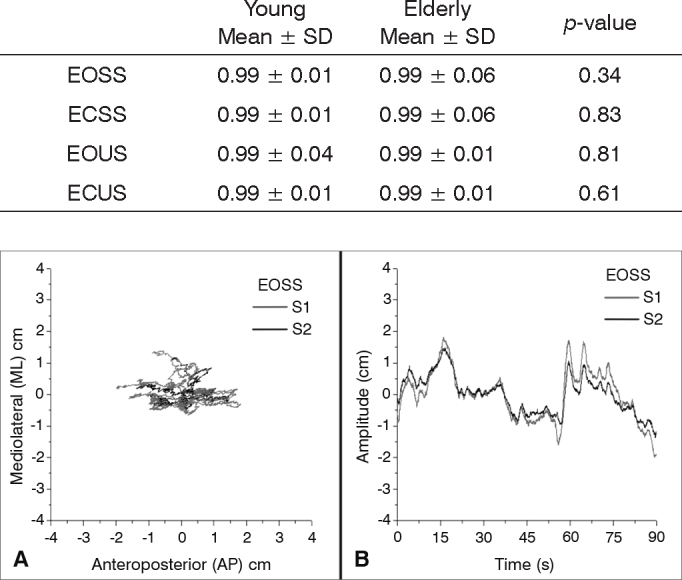
Statokinesigram (A) and Stabilogram (B) of a young subject registered by sensor 1 (S1) and sensor 2 (S2) under conditions of eyes open stable surface (EOSS).

Figure 2A shows the trajectory of AP postural oscillation versus ML direction of a young subject in the EOSS condition. The postural oscillation trajectory of an individual during the 90 s registration time is shown for the two axis and characterizes the graph of postural oscillation in the AP versus ML axis. In analogy with the force platform graphs, this plot was named statokinesigram.

Figure 2B shows the displacement of the same subject in the EOSS condition. In this plot, it is possible to observe the amplitude of postural oscillation in relation to time in the AP axis and the high concordance of the sensor movements is clearly demonstrated. In analogy with the force platform graphs, this plot was named stabilogram.

## DISCUSSION

In this study, the electromagnetic sensor system employed was shown to be efficient for oscillation analysis since it delivered detailed information about the kinematics of the body segments and, therefore, about postural balance^17^. In 1989, Pearcy and Hindle demonstrated that an electromagnetic tracking system could provide high resolution accuracy and repeatability in spinal motion analysis^18^. The use of this equipment in oscillation analysis is still little known. We developed a user-friendly and easy-to-perform software for the analysis of human posture that organizes and analyzes the acquired data.

The position of the sensors is essential for the characterization of the different types of postural oscillations. In this study, one sensor was positioned on the sacral region (between the S2 and S3 vertebrae) in order to be close to the body mass center and to allow the interpretation of the position of the ankle, since the subjects were asked to keep their knees in extension. The other sensor was placed on the posterior thoracic region because this is the most fixed region of the spine and, therefore, the registered movements would correctly represent the trunk oscillation. Also, with the sensors positioned in this manner it was possible to verify the concordance between these segments and to characterize oscillations in inverted pendulum (ankle strategy) or double inverted pendulum (hip strategy). Although the isolated ante and retroversion movement of the hip is not described as a relevant point in the literature, we performed an additional analysis of this aspect. This movement was not observed in the present volunteers.

Accornero et al.^11^ also employed electromagnetic sensors to study the agreement of body segments in the static position in young and older adults. In that study, however, the magnetic sensors were positioned on the head and lumbar region. The lumbar region is one of the most flexible structures of the human spine, a fact that impairs the interpretation of hip and ankle oscillations. The same interpretation applies to the head, which moves with many degrees of freedom. In principle, the hip and ankle strategy could be estimated using two sensors and the joints that should be analyzed in these models correspond to the ankle and hip^19^. Since some patients can move both the head and lumbar region without hip motion, the positions of the sensor chosen by Accornero et al.^11^ cannot always reflect the postural strategies. Therefore, the study of Accornero et al. was important by showing the oscillation of the head in relation to the trunk but not for identifying different postural strategies. Thus, it is more proper to position the sensor over the thoracic vertebra and the sacral region in order to observe the flexibility of the hip joint and, consequently, the postural strategy.

During the tests under different sensory conditions, also known as modified Clinical Test of Sensory Interaction on Balance (mCTSIB), when the influence of vision on postural oscillation was analyzed, we observed that the influence of visual afferences was more important on the unstable surface than on the stable surface. When the influence of different proprioceptive conditions was analyzed, there was more instability on the unstable surface with eyes both open and closed. Studies using the force platform to measure the center of pressure in young and elderly healthy volunteers also showed more postural oscillation when some information was reduced or withdrawn^20^, ^21^, ^22^, ^23^.

In the present study no differences were observed between young (men and women) in different sensory conditions, in agreement with previous studies, which also did not detect differences between genders^21^, ^22^. The concordance of the results of this study with previous ones indicates the importance of the proposed technique for postural oscillation analysis in different sensory conditions.

In order to identify the agreement of the body segments, that is, the relation between ankle/hip/trunk, we determined the concordance of sensors 1 and 2 along the AP axis using cross-correlation analysis. In all situations the values obtained were very high (near +1), indicating concordance of the postural oscillation of the different body segments and characterizing the inverted pendulum strategy. This high cross-correlation between sensors 1 and 2 indicated that the control of static balance in the AP direction in young and elderly people is mainly performed by the ankle musculature (plantarflexors/dorsiflexors) in all of the sensory conditions analyzed in the present study. No significant differences in this parameter were detected between different sensory conditions or between groups.

In contrast, Accornero et al.^11^ reported higher postural rigidity with eyes open and closed in healthy subjects. Nevertheless, as described above, there are significant methodological differences between the latter study and the present one. As discussed by Colobert et al.^24^, calculated strategies during quiet stance must be interpreted with care. Since the presentation of multisegmental coordination by Nashner & McCollum^8^ and Nashner^25^, ankle and hip strategies have been differentiated on the basis of muscle activity, joint movement and forces generated by postural activity with respect to the support surface.

However, to the best of our knowledge, a threshold score to differentiate those strategies has not yet been described. Most of the studies consider patterns of postural oscillation and compare the statistical results between two groups and conclude that one group predominantly presents hip or ankle strategy. In this context, Varoqui et al.^26^, used four electrogoniometers fixed to the ankles and the hips and they considered two ankle-hip postural patterns - 0° (in-phase) and 180° (anti-phase). Liaw et al.^27^ and Lee et al.^28^, using force platforms considered that the maximal stability score of 100% implied the highest stability, while a score of 0% implied the least stability. The ankle strategy scores ranged from 0% to 100%. A score of 100% implied a predominance of ankle strategy and 0% implied a predominance of hip strategy. Also, Termoz et al.^16^ analyzed center of pressure and center of mass data to calculate cross-correlations in the A/P direction. In the present study, two electromagnetic sensors were fixated in the sacral and thoracic regions and the cross-correlation was calculated. Certainly, the high cross-correlation results observed in the subjects represent the ankle strategy. Nevertheless, as the joint motion in the quiet stance condition is never a single strategy, it is important to determine a threshold value that can be used to determine the strategy employed in large and different groups of subjects. An additional comparison of the data provided by the electromagnetic system with other methods should also be performed in order to validate the method that has been proposed.

Although it is more proper to perform at least three trials^29^, several studies also conducted only one trial^11^, ^30^, ^31^, ^32^, ^33^. Moreover, using 90 s to collect data instead of 60 s as done in many previous studies increases the sensitivity of the present data. Thus, the methodology of the present study can be considered representative, reliable and comparable with previous studies. A limitation of the use of this system is the site for data acquisition, since it is necessary to avoid places with a considerable amount of metal in their structure or that may cause a magnetic field capable of directly interfering with data collection. However, a great advantage over the force platform is it the facility of transport to different locations 11, 17, 34, 35.

Furthermore, the electromagnetic system has several clinical applications including the analysis of general physical activity, gait, posture, trunk and upper limb mo-vement^17^. This tool may become useful for helping define appropriate rehabilitation measures and to provide important information to be used when monitoring the results of any given therapy^35^. The specific analysis of postural strategy with the methodology used in the present study allows the investigation of several populations and has proved to be technically reliable, affordable, and effective. Adding a stimulus that causes visual conflict and using a moving surface to evaluate the dynamics of postural control may significantly increase the scope of this device. Also, this system permits the inclusion of more than two sensors, which can enhance the kinematic analysis of postural control.

## CONCLUSION

This study presented an important apparatus for the evaluation of the postural strategies of young and elderly subjects in different sensory conditions, with the sensors fixed in the spinous process of the first thoracic vertebra and over the sacral region. Both young and elderly subjects presented a strong correlation between the sensors (ankle strategy), with no differences between the groups.

## COMPETING INTERESTS

The authors declare that they have no competing interests.

## AUTHORS' CONTRIBUTIONS

JAOC, TEGSP participated in the design of the study, performed the statistical analysis and drafted the manuscript. JFC made substantial contributions to data analysis and interpretation. AAOC participated in the design of the study and made substantial contributions to data analysis and interpretation. EF helped with the draft of the manuscript and made substantial contributions. All authors read and approved the final manuscript.

## ACKNOWLEDGEMENTS

Research supported in part by FAEPA and CAPES.
